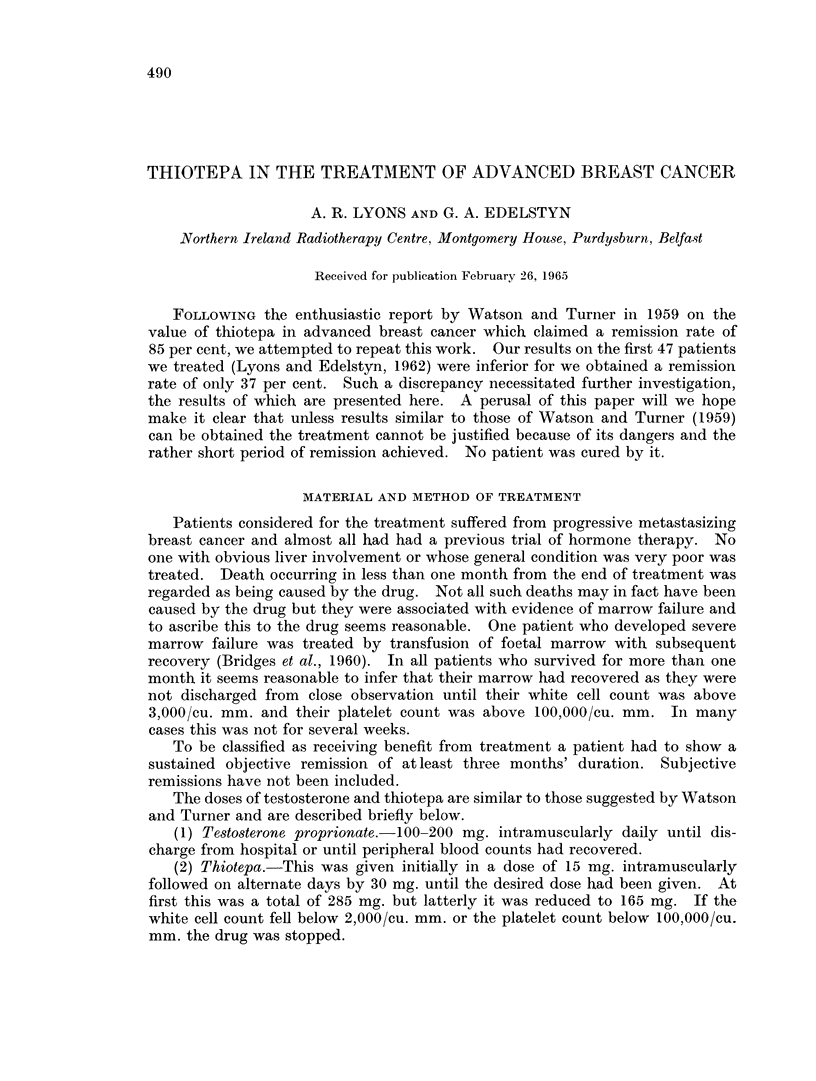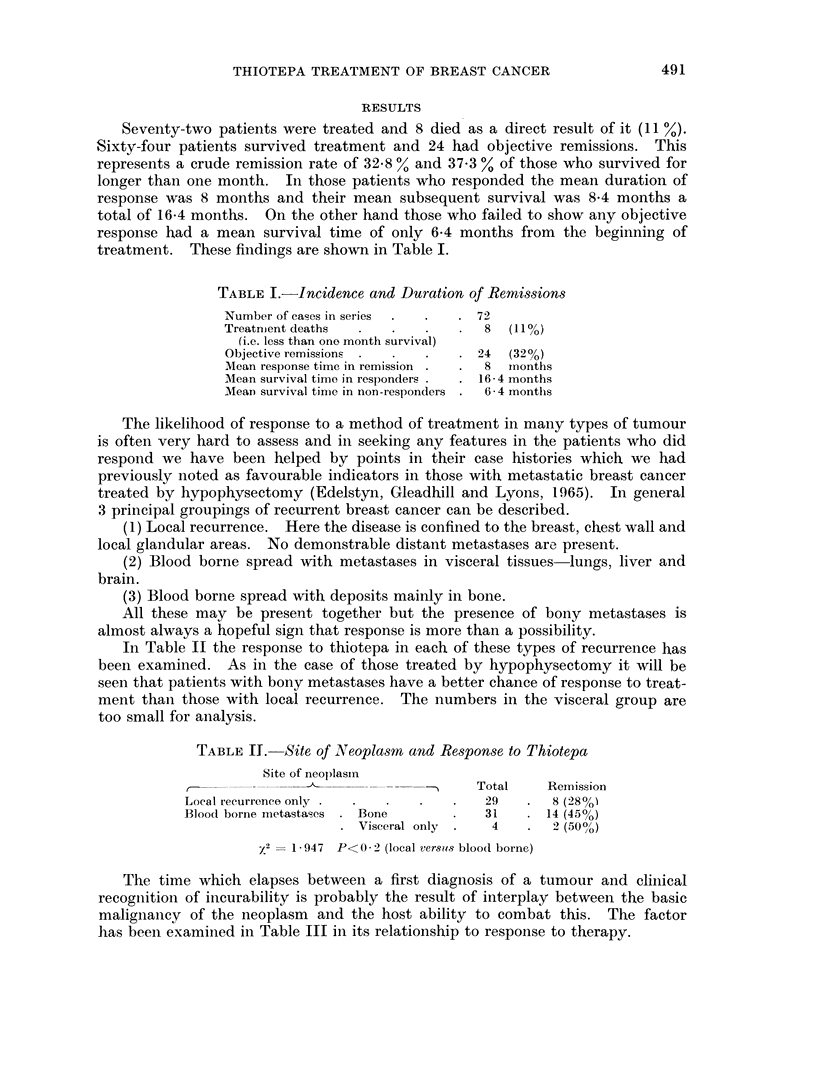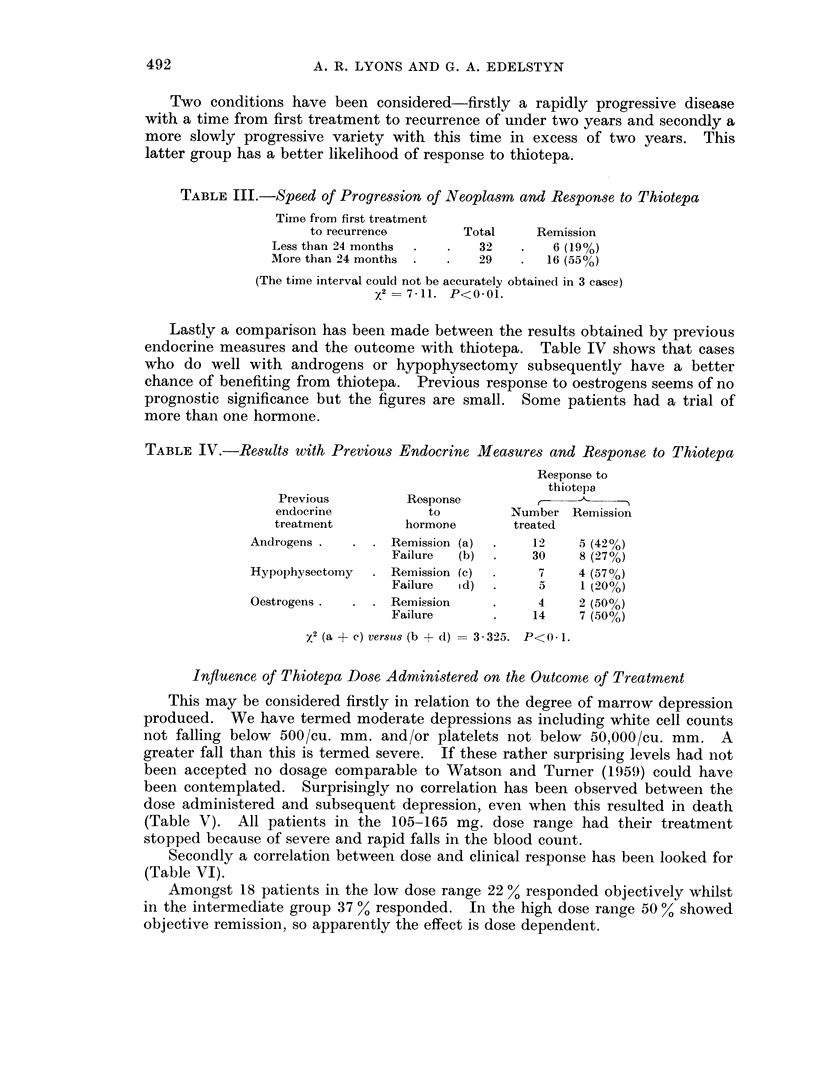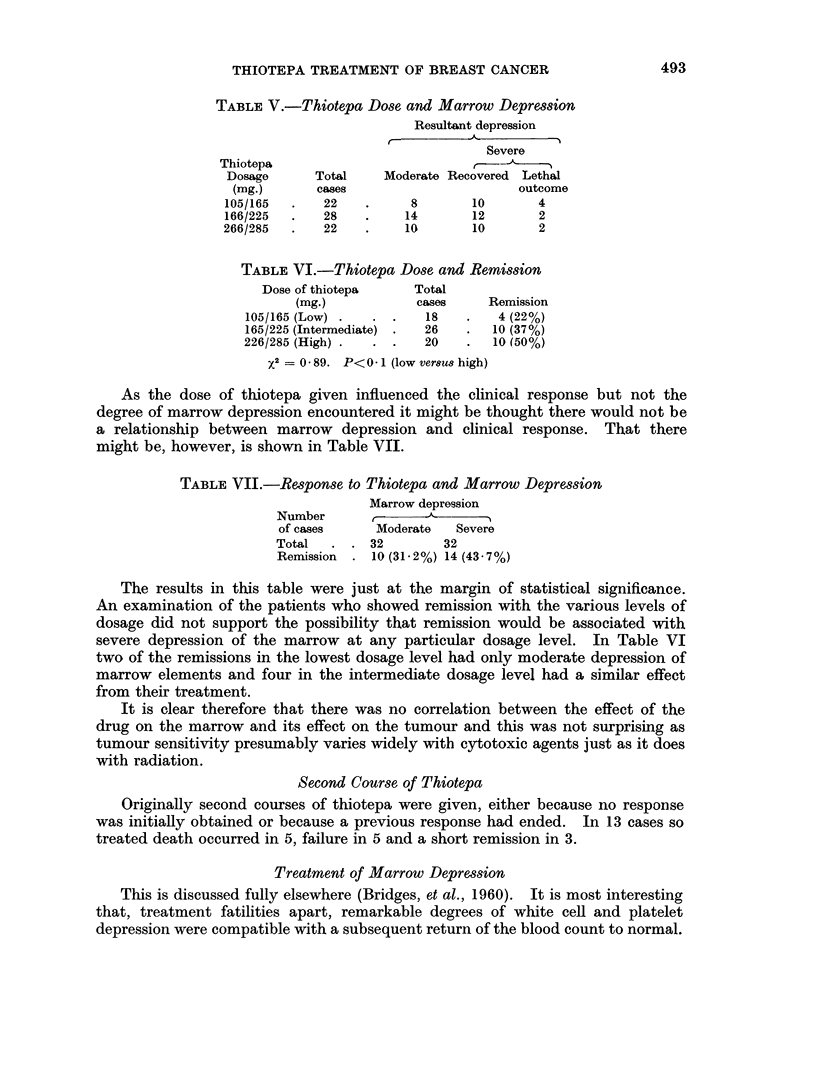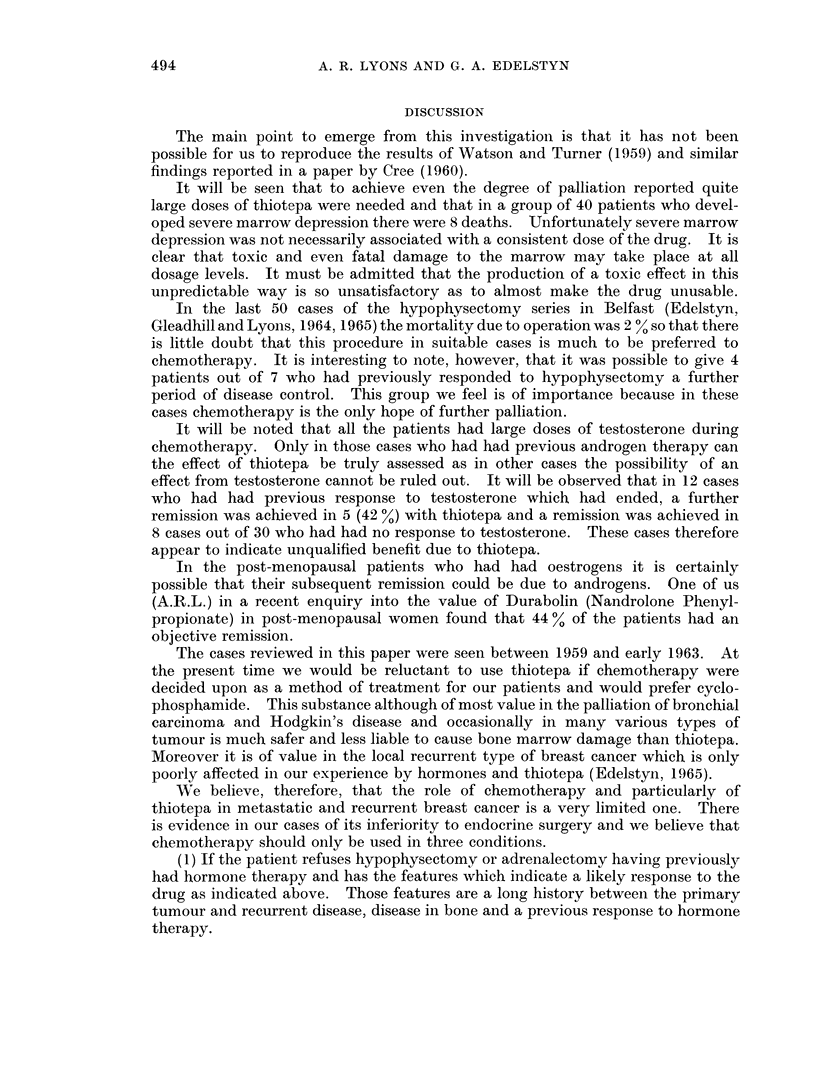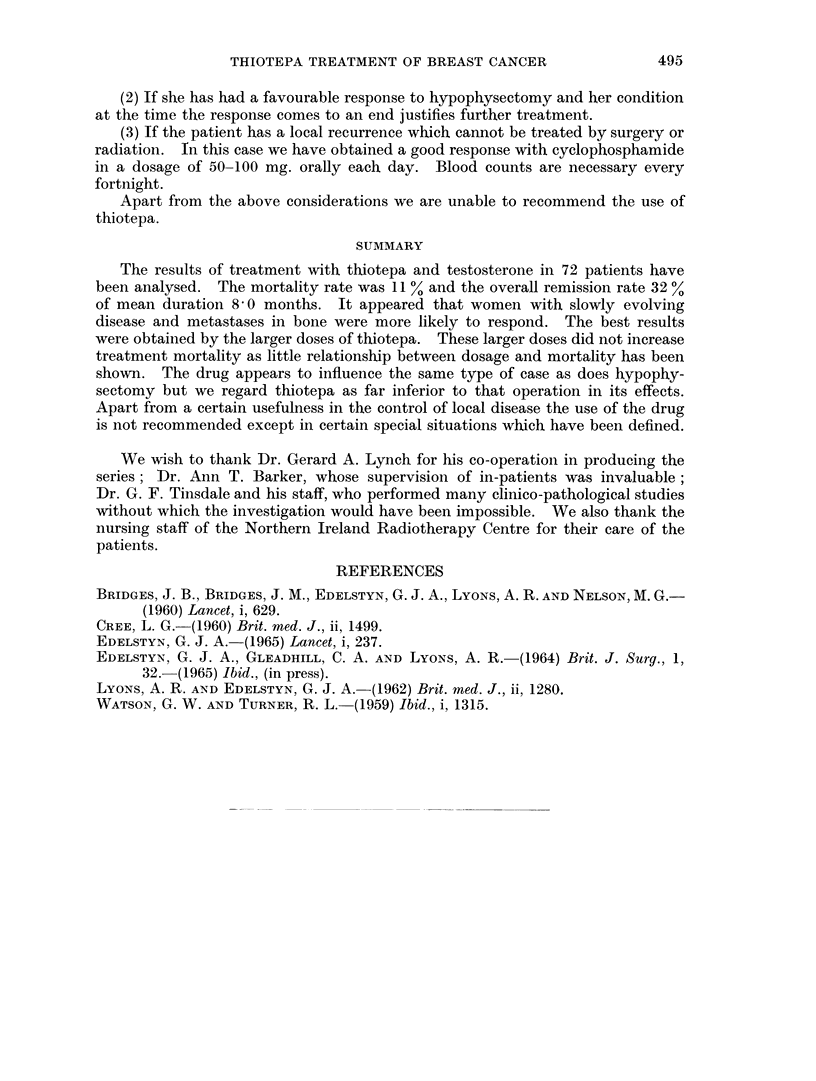# Thiotepa in the treatment of advanced breast cancer.

**DOI:** 10.1038/bjc.1965.58

**Published:** 1965-09

**Authors:** A. R. Lyons, G. A. Edelstyn


### THIOTEPA IN THE TREATMENT OF ADVANCED BREAST CANCER

A. R. LYONS AND G. A. EDELSTYN

*Northern Ireland Radiotherapy Centre, Montgomery House, Purdysburn, Belfast*

Received for publication February 26, 1965

FOLLOWING the enthusiastic report by Watson and Turner in 1959 on the value of thiotepa in advanced breast cancer which claimed a remission rate of 85 per cent, we attempted to repeat this work. Our results on the first 47 patients we treated (Lyons and Edelstyn, 1962) were inferior for we obtained a remission rate of only 37 per cent. Such a discrepancy necessitated further investigation, the results of which are presented here. A perusal of this paper will we hope make it clear that unless results similar to those of Watson and Turner (1959) can be obtained the treatment cannot be justified because of its dangers and the rather short period of remission achieved. No patient was cured by it.

### MATERIAL AND METHOD OF TREATMENT

Patients considered for the treatment suffered from progressive metastasizing breast cancer and almost all had had a previous trial of hormone therapy. No one with obvious liver involvement or whose general condition was very poor was treated. Death occurring in less than one month from the end of treatment was regarded as being caused by the drug. Not all such deaths may in fact have been caused by the drug but they were associated with evidence of marrow failure and to ascribe this to the drug seems reasonable. One patient who developed severe marrow failure was treated by transfusion of foetal marrow with subsequent recovery (Bridges *et al.*, 1960). In all patients who survived for more than one month it seems reasonable to infer that their marrow had recovered as they were not discharged from close observation until their white cell count was above 3,000/cu. mm. and their platelet count was above 100,000/cu. mm. In many cases this was not for several weeks.

To be classified as receiving benefit from treatment a patient had to show a sustained objective remission of at least three months' duration. Subjective remissions have not been included.

The doses of testosterone and thiotepa are similar to those suggested by Watson and Turner and are described briefly below.

(1) *Testosterone proprionate.*—100–200 mg. intramuscularly daily until discharge from hospital or until peripheral blood counts had recovered.

(2) *Thiotepa.*—This was given initially in a dose of 15 mg. intramuscularly followed on alternate days by 30 mg. until the desired dose had been given. At first this was a total of 285 mg. but latterly it was reduced to 165 mg. If the white cell count fell below 2,000/cu. mm. or the platelet count below 100,000/cu. mm. the drug was stopped.

### RESULTS

Seventy-two patients were treated and 8 died as a direct result of it (11 %). Sixty-four patients survived treatment and 24 had objective remissions. This represents a crude remission rate of 32·8 % and 37·3 % of those who survived for longer than one month. In those patients who responded the mean duration of response was 8 months and their mean subsequent survival was 8·4 months a total of 16·4 months. On the other hand those who failed to show any objective response had a mean survival time of only 6·4 months from the beginning of treatment. These findings are shown in Table I.

TABLE I.—*Incidence and Duration of Remissions*

| | | |
|---|---|---|
| Number of cases in series | 72 | |
| Treatment deaths (i.e. less than one month survival) | 8 | (11%) |
| Objective remissions | 24 | (32%) |
| Mean response time in remission | 8 | months |
| Mean survival time in responders | 16·4 | months |
| Mean survival time in non-responders | 6·4 | months |

The likelihood of response to a method of treatment in many types of tumour is often very hard to assess and in seeking any features in the patients who did respond we have been helped by points in their case histories which we had previously noted as favourable indicators in those with metastatic breast cancer treated by hypophysectomy (Edelstyn, Gleadhill and Lyons, 1965). In general 3 principal groupings of recurrent breast cancer can be described.

(1) Local recurrence. Here the disease is confined to the breast, chest wall and local glandular areas. No demonstrable distant metastases are present.

(2) Blood borne spread with metastases in visceral tissues—lungs, liver and brain.

(3) Blood borne spread with deposits mainly in bone.

All these may be present together but the presence of bony metastases is almost always a hopeful sign that response is more than a possibility.

In Table II the response to thiotepa in each of these types of recurrence has been examined. As in the case of those treated by hypophysectomy it will be seen that patients with bony metastases have a better chance of response to treatment than those with local recurrence. The numbers in the visceral group are too small for analysis.

TABLE II.—*Site of Neoplasm and Response to Thiotepa*

| Site of neoplasm | | Total | Remission |
|---|---|---|---|
| Local recurrence only | | 29 | 8 (28%) |
| Blood borne metastases | Bone | 31 | 14 (45%) |
| | Visceral only | 4 | 2 (50%) |

$\chi^2 = 1 \cdot 947$ $P < 0 \cdot 2$ (local *versus* blood borne)

The time which elapses between a first diagnosis of a tumour and clinical recognition of incurability is probably the result of interplay between the basic malignancy of the neoplasm and the host ability to combat this. The factor has been examined in Table III in its relationship to response to therapy.

Two conditions have been considered—firstly a rapidly progressive disease with a time from first treatment to recurrence of under two years and secondly a more slowly progressive variety with this time in excess of two years. This latter group has a better likelihood of response to thiotepa.

TABLE III.—*Speed of Progression of Neoplasm and Response to Thiotepa*

| Time from first treatment to recurrence | Total | Remission |
|---|---|---|
| Less than 24 months . . | 32 . | 6 (19%) |
| More than 24 months . . | 29 . | 16 (55%) |

(The time interval could not be accurately obtained in 3 cases)
$\chi^2 = 7 \cdot 11$. $P < 0 \cdot 01$.

Lastly a comparison has been made between the results obtained by previous endocrine measures and the outcome with thiotepa. Table IV shows that cases who do well with androgens or hypophysectomy subsequently have a better chance of benefiting from thiotepa. Previous response to oestrogens seems of no prognostic significance but the figures are small. Some patients had a trial of more than one hormone.

TABLE IV.—*Results with Previous Endocrine Measures and Response to Thiotepa*

| Previous endocrine treatment | Response to hormone | Response to thiotepa: Number treated | Remission |
|---|---|---|---|
| Androgens . . . | Remission (a) . | 12 | 5 (42%) |
| | Failure (b) . | 30 | 8 (27%) |
| Hypophysectomy . | Remission (c) . | 7 | 4 (57%) |
| | Failure (d) . | 5 | 1 (20%) |
| Oestrogens . . . | Remission . | 4 | 2 (50%) |
| | Failure . | 14 | 7 (50%) |

$\chi^2$ (a + c) *versus* (b + d) = $3 \cdot 325$. $P < 0 \cdot 1$.

*Influence of Thiotepa Dose Administered on the Outcome of Treatment*

This may be considered firstly in relation to the degree of marrow depression produced. We have termed moderate depressions as including white cell counts not falling below 500/cu. mm. and/or platelets not below 50,000/cu. mm. A greater fall than this is termed severe. If these rather surprising levels had not been accepted no dosage comparable to Watson and Turner (1959) could have been contemplated. Surprisingly no correlation has been observed between the dose administered and subsequent depression, even when this resulted in death (Table V). All patients in the 105–165 mg. dose range had their treatment stopped because of severe and rapid falls in the blood count.

Secondly a correlation between dose and clinical response has been looked for (Table VI).

Amongst 18 patients in the low dose range 22 % responded objectively whilst in the intermediate group 37 % responded. In the high dose range 50 % showed objective remission, so apparently the effect is dose dependent.

TABLE V.—*Thiotepa Dose and Marrow Depression*

| Thiotepa Dosage (mg.) | Total cases | Resultant depression: Moderate | Severe: Recovered | Severe: Lethal outcome |
|---|---|---|---|---|
| 105/165 | 22 | 8 | 10 | 4 |
| 166/225 | 28 | 14 | 12 | 2 |
| 266/285 | 22 | 10 | 10 | 2 |

TABLE VI.—*Thiotepa Dose and Remission*

| Dose of thiotepa (mg.) | Total cases | Remission |
|---|---|---|
| 105/165 (Low) | 18 | 4 (22%) |
| 165/225 (Intermediate) | 26 | 10 (37%) |
| 226/285 (High) | 20 | 10 (50%) |

$\chi^2 = 0 \cdot 89$. $P < 0 \cdot 1$ (low *versus* high)

As the dose of thiotepa given influenced the clinical response but not the degree of marrow depression encountered it might be thought there would not be a relationship between marrow depression and clinical response. That there might be, however, is shown in Table VII.

TABLE VII.—*Response to Thiotepa and Marrow Depression*

| Number of cases | Marrow depression: Moderate | Severe |
|---|---|---|
| Total | 32 | 32 |
| Remission | 10 (31·2%) | 14 (43·7%) |

The results in this table were just at the margin of statistical significance. An examination of the patients who showed remission with the various levels of dosage did not support the possibility that remission would be associated with severe depression of the marrow at any particular dosage level. In Table VI two of the remissions in the lowest dosage level had only moderate depression of marrow elements and four in the intermediate dosage level had a similar effect from their treatment.

It is clear therefore that there was no correlation between the effect of the drug on the marrow and its effect on the tumour and this was not surprising as tumour sensitivity presumably varies widely with cytotoxic agents just as it does with radiation.

### *Second Course of Thiotepa*

Originally second courses of thiotepa were given, either because no response was initially obtained or because a previous response had ended. In 13 cases so treated death occurred in 5, failure in 5 and a short remission in 3.

### *Treatment of Marrow Depression*

This is discussed fully elsewhere (Bridges, *et al.*, 1960). It is most interesting that, treatment fatilities apart, remarkable degrees of white cell and platelet depression were compatible with a subsequent return of the blood count to normal.

### DISCUSSION

The main point to emerge from this investigation is that it has not been possible for us to reproduce the results of Watson and Turner (1959) and similar findings reported in a paper by Cree (1960).

It will be seen that to achieve even the degree of palliation reported quite large doses of thiotepa were needed and that in a group of 40 patients who developed severe marrow depression there were 8 deaths. Unfortunately severe marrow depression was not necessarily associated with a consistent dose of the drug. It is clear that toxic and even fatal damage to the marrow may take place at all dosage levels. It must be admitted that the production of a toxic effect in this unpredictable way is so unsatisfactory as to almost make the drug unusable.

In the last 50 cases of the hypophysectomy series in Belfast (Edelstyn, Gleadhill and Lyons, 1964, 1965) the mortality due to operation was 2 % so that there is little doubt that this procedure in suitable cases is much to be preferred to chemotherapy. It is interesting to note, however, that it was possible to give 4 patients out of 7 who had previously responded to hypophysectomy a further period of disease control. This group we feel is of importance because in these cases chemotherapy is the only hope of further palliation.

It will be noted that all the patients had large doses of testosterone during chemotherapy. Only in those cases who had had previous androgen therapy can the effect of thiotepa be truly assessed as in other cases the possibility of an effect from testosterone cannot be ruled out. It will be observed that in 12 cases who had had previous response to testosterone which had ended, a further remission was achieved in 5 (42 %) with thiotepa and a remission was achieved in 8 cases out of 30 who had had no response to testosterone. These cases therefore appear to indicate unqualified benefit due to thiotepa.

In the post-menopausal patients who had had oestrogens it is certainly possible that their subsequent remission could be due to androgens. One of us (A.R.L.) in a recent enquiry into the value of Durabolin (Nandrolone Phenylpropionate) in post-menopausal women found that 44 % of the patients had an objective remission.

The cases reviewed in this paper were seen between 1959 and early 1963. At the present time we would be reluctant to use thiotepa if chemotherapy were decided upon as a method of treatment for our patients and would prefer cyclophosphamide. This substance although of most value in the palliation of bronchial carcinoma and Hodgkin's disease and occasionally in many various types of tumour is much safer and less liable to cause bone marrow damage than thiotepa. Moreover it is of value in the local recurrent type of breast cancer which is only poorly affected in our experience by hormones and thiotepa (Edelstyn, 1965).

We believe, therefore, that the role of chemotherapy and particularly of thiotepa in metastatic and recurrent breast cancer is a very limited one. There is evidence in our cases of its inferiority to endocrine surgery and we believe that chemotherapy should only be used in three conditions.

(1) If the patient refuses hypophysectomy or adrenalectomy having previously had hormone therapy and has the features which indicate a likely response to the drug as indicated above. Those features are a long history between the primary tumour and recurrent disease, disease in bone and a previous response to hormone therapy.

(2) If she has had a favourable response to hypophysectomy and her condition at the time the response comes to an end justifies further treatment.

(3) If the patient has a local recurrence which cannot be treated by surgery or radiation. In this case we have obtained a good response with cyclophosphamide in a dosage of 50–100 mg. orally each day. Blood counts are necessary every fortnight.

Apart from the above considerations we are unable to recommend the use of thiotepa.

### SUMMARY

The results of treatment with thiotepa and testosterone in 72 patients have been analysed. The mortality rate was 11 % and the overall remission rate 32 % of mean duration 8·0 months. It appeared that women with slowly evolving disease and metastases in bone were more likely to respond. The best results were obtained by the larger doses of thiotepa. These larger doses did not increase treatment mortality as little relationship between dosage and mortality has been shown. The drug appears to influence the same type of case as does hypophysectomy but we regard thiotepa as far inferior to that operation in its effects. Apart from a certain usefulness in the control of local disease the use of the drug is not recommended except in certain special situations which have been defined.

We wish to thank Dr. Gerard A. Lynch for his co-operation in producing the series ; Dr. Ann T. Barker, whose supervision of in-patients was invaluable ; Dr. G. F. Tinsdale and his staff, who performed many clinico-pathological studies without which the investigation would have been impossible. We also thank the nursing staff of the Northern Ireland Radiotherapy Centre for their care of the patients.

### REFERENCES

Bridges, J. B., Bridges, J. M., Edelstyn, G. J. A., Lyons, A. R. and Nelson, M. G.—(1960) *Lancet*, i, 629.

Cree, L. G.—(1960) *Brit. med. J.*, ii, 1499.

Edelstyn, G. J. A.—(1965) *Lancet*, i, 237.

Edelstyn, G. J. A., Gleadhill, C. A. and Lyons, A. R.—(1964) *Brit. J. Surg.*, 1, 32.—(1965) *Ibid.*, (in press).

Lyons, A. R. and Edelstyn, G. J. A.—(1962) *Brit. med. J.*, ii, 1280.

Watson, G. W. and Turner, R. L.—(1959) *Ibid.*, i, 1315.